# Synergistic activity of dispersin B and benzoyl peroxide against *Cutibacterium acnes*/*Staphylococcus epidermidis* dual-species biofilms

**DOI:** 10.1371/journal.pone.0320662

**Published:** 2025-03-27

**Authors:** Jeffrey B. Kaplan, Anna Muzaleva, Miloslav Sailer, Robert B. Huizinga, Khalaf Kridin

**Affiliations:** 1 Department of Biology, American University, Washington, District of Columbia, United States of America; 2 Laboratory for Skin Research, Institute for Medical Research, Galilee Medical Center, Nahariya, Israel; 3 Kane Biotech, Inc., Winnipeg, Manitoba, Canada; 4 The Azrieli Faculty of Medicine, Bar-Ilan University, Safed, Israel; Cairo University - Faculty of Pharmacy, EGYPT

## Abstract

*Cutibacterium acnes* plays a key role in the development of acne vulgaris, with biofilm formation contributing to its persistence and resistance to antimicrobial treatments. A critical component of *C. acnes* biofilms is poly-*N*-acetylglucosamine (PNAG), an exopolysaccharide that facilitates both biofilm stability and biocide resistance. This study evaluated the efficacy of the PNAG-degrading enzyme dispersin B in enhancing the susceptibility of *C. acnes* biofilms to benzoyl peroxide (BP), a common anti-acne agent. Dual-species biofilms of *C. acnes* and *Staphylococcus epidermidis*, which has been shown to promote *C. acnes* biofilm growth under aerobic conditions, were cultivated in glass tubes and treated with dispersin B (5–80 µg/mL), BP (0.1–2.5%), or a combination of both. Dispersin B or BP alone reduced *C. acnes* colony-forming units (CFUs) by 1–2 log units. However, sequential treatment with dispersin B followed by BP achieved a synergistic effect, yielding a >6-log reduction in CFUs. Remarkably, concentrations as low as 5 µg/mL dispersin B combined with 0.5% BP efficiently eradicated *C. acnes* from the dual-species biofilms. These findings highlight the protective role of PNAG against BP and demonstrate the potential of dispersin B as an adjunctive therapy to enhance the efficacy of BP in acne treatment.

## 1. Introduction

The Gram-positive bacterium *Cutibacterium acnes* was first isolated from a patient with acne vulgaris more than 100 years ago [1]. *C. acnes* is an aerotolerant anaerobe that utilizes sebum and keratin as nutrient sources, making it well adapted for life inside hair follicles. Although some *C. acnes* cellular components such as porphyrins, surface proteins, cytotoxic exoenzymes, and biofilms have been shown to trigger inflammation in the later stages of acne [2], the precise role of *C. acnes* in the early stages of acne remains unknown. For example, *C. acnes* colonizes hair follicles in all people, and several previous studies found no difference in the abundance of *C. acnes* in subjects with or without acne [3,4]. Recent studies suggest that acne might be the result of an unbalanced equilibrium between *C. acnes* and *Staphylococcus epidermidis*, another major skin resident [3,5–9]. In one study that identified bacteria in hair follicles from acne patients and healthy controls using a highly sensitive cyanoacrylate biopsy method [10], all subjects harbored *C. acnes* within their follicles, but the follicles of acne patients also harbored *S. epidermidis*.

Biofilms are defined as densely packed layers of bacterial cells growing attached to a tissue or surface [11]. Bacteria in a biofilm are encased in a sticky, self-synthesized, extracellular polymeric matrix that holds the cells together in a mass, attaches them to the underlying surface, and protects them from killing by antimicrobial agents and host immunity. Biofilms play a role in many chronic infections which are often difficult to treat because of the protective nature of biofilms. *In vivo*, *C. acnes* biofilms have been observed in acne lesions [12] and *S. epidermidis* biofilms have been observed on skin and in sweat glands [13]. Both species have been shown to form biofilms *in vitro* [1,14] and on the surfaces of implanted medical devices *in vivo* [15,16].

Poly-*N*-acetylglucosamine (PNAG) is an extracellular polysaccharide that mediates biofilm formation, antimicrobial resistance, host colonization, immune evasion, and stress tolerance in a wide range of bacterial and fungal pathogens [17,18]. PNAG is an essential virulence factor for *Staphylococcus aureus* in mouse models of systemic infection [19]; for *Aggregatibacter actinomycetemcomitans* in a rat model of periodontitis [20]; for *Klebsiella pneumoniae* in a mouse model of intestinal colonization and systemic infection [21]; and for *Actinobacillus pleuropneumoniae* during natural porcine pleuropneumonia infection in pigs [22]. Anti-PNAG antibodies have been shown to protect mice against local and/or systemic infections caused by *Streptococcus pyogenes*, *Streptococcus pneumoniae*, *Listeria monocytogenes*, *Neisseria meningitidis* serogroup B, and *Candida albicans* [17], suggesting that PNAG is an important virulence factor in these organisms. Previous studies showed that both *C. acnes* and *S. epidermidis* produce PNAG [23,24].

Dispersin B is an enzyme that hydrolyzes PNAG [25]. Dispersin B has been shown to inhibit the attachment of *C. acnes* cells to surfaces, to inhibit *C. acnes* biofilm formation, and to sensitize *C. acnes* biofilms to killing by tetracycline and the common anti-acne agent benzoyl peroxide (BP) *in vitro* [24]. Dispersin B has also been shown and to inhibit *S. epidermidis* biofilm formation, detach pre-formed *S. epidermidis* biofilms, and sensitize pre-formed *S. epidermidis* biofilm cells to killing by cetylpyridinum chloride and rifampicin *in vitro* [26,27], and to inhibit attachment of *S. epidermidis* cells to pig skin *in vivo* [28].

While *C. acnes* is an anaerobe whose growth is inhibited by oxygen, previous studies showed that *C. acnes* can form robust biofilms in air when co-cultured with *S. epidermidis* [29]. This suggests that aerobic *C. acnes*/*S. epidermidis* dual-species biofilms may mimic biofilm formation in the skin’s microenvironments, including the well-oxygenated dermis and the hypoxic but non-anoxic conditions of sebaceous glands and hair follicles [30]. However, the structural components supporting these biofilms and their resistance to antimicrobial agents remain poorly understood. PNAG is a known adhesin in *C. acnes* and *S. epidermidis* biofilms, yet its role in maintaining dual-species biofilms under aerobic conditions has not been investigated. Furthermore, while dispersin B has been shown to disrupt single-species biofilms of *C. acnes* and *S. epidermidis*, its impact on dual-species biofilms and their susceptibility to BP remains unclear. To address this gap, the present study tested the hypothesis that PNAG is a crucial structural component of aerobic *C. acnes*/*S. epidermidis* dual-species biofilms and that enzymatic degradation of PNAG by dispersin B would enhance BP-mediated killing.

## 2. Materials and methods

### 2.1. Bacterial strains

The bacterial strains used in this study were *C. acnes* HL086PA1 [31] and *S. epidermidis* strain 5 [32]. *C. acnes* HL086PA1, an erythromycin-resistant strain isolated from a patient with severe acne, was obtained from BEI Resources (Manassas, Virginia, USA) as part of the NIAID NIH Human Microbiome Project. *S. epidermidis* strain 5 is an erythromycin susceptible, PNAG-producing strain isolated from an implant infection that was previously shown to facilitate growth of *C. acnes* HL086PA1 biofilms *in vitro* [29]. The media used were Tryptic Soy agar (TSA) and Tryptic Soy broth (TSB), both purchased in powder form from Becton, Dickinson and Company (BD; Franklin Lakes, New Jersey, USA). All bacteria were cultured at 37 °C. Reference stock cultures were maintained at −80 °C in 20% glycerol/80% TSB. Working stock cultures were restreaked weekly on TSA. *S. epidermidis* working stocks were incubated for 1 d in air. *C. acnes* working stocks were incubated for 3 d under anaerobic conditions generated with a BD GasPak EZ Anaerobe sachet system.

### 2.2. Biofilm culture

Inocula for biofilm cultures were prepared by transferring a 1- µL-size loopful of *S. epidermidis* cells from a 24-h-old agar plate into 200 µL of phosphate buffered saline (PBS), and then transferring a loopful of *C. acnes* cells from 72-h-old agar plate into the same tube. The cells were mixed by vortex agitation, diluted 1:1,000 in filter sterilized TSB, and then passed through a 5-µm pore-size syringe filter to remove large clumps of cells. Diluted inocula contained 10^6^–10^7^ CFU/mL of each species. Bacteria were present at a *S. epidermidis*:*C. acnes* ratio of 2–10:1. Filtered inocula were aliquoted into sterile 13 ×  100 mm glass tubes (1 mL/tube) and incubated for 24 h statically at 37 °C in air.

### 2.3. Dispersin B

Two formulations of dispersin B were employed. The first was a dispersin B solution which contained 1 mg/mL recombinant dispersin B protein dissolved in 50 mM phosphate buffer (pH 5.8), 100 mM NaCl, and 50% glycerol. Dispersin B solution was diluted in PBS to achieve a working concentration of 80 µg/mL enzyme. The second formulation was a dispersin B hydrogel which contained 80 µg/mL dispersin B in a mixture of non-ionic surfactant (poloxamer 407), glycerol, levulininc acid, anisic acid, and phosphate buffer. The dispersin B hydrogel was used directly or diluted with blank hydrogel (no enzyme) to achieve working concentrations of 40, 20, 10 and 5 µg/mL of enzyme. Dispersin B solution, dispersin B hydrogel, and blank hydrogel were provided by Kane Biotech (Winnipeg, Manitoba, Canada).

### 2.4. Benzoyl peroxide

CVS Health brand Maximum Strength Acne Treatment Gel containing 10% benzoyl peroxide (BP) was employed (CVS Corporation, Woonsocket, Rhode Island, USA). This gel contains micronized BP particles evenly dispersed within a polymer-based gelling agent. Although BP has extremely low solubility in water (~0.001%), its effectiveness at higher concentrations in gel formulations is due to its use as a suspension rather than a solution. The gel was diluted in PBS to obtain final working concentrations of 2.5%, 1%, 0.5%, and 0.1% BP.

### 2.5. Two-step biofilm treatment and enumeration of biofilm CFUs

After growth of biofilms for 24 h, the media was gently aspirated using a finely drawn Pasteur pipette. Each tube was rinsed twice with 3 mL of PBS and gently aspirated after each rinse. For the first treatment, tubes were filled with 1 mL of PBS; 80 µg/mL dispersin B solution; dispersin B hydrogel containing 5, 10, 20, 40 or 80 µg/mL dispersin B; or 1% or 2.5% BP, as indicated. Tubes were incubated for 15 min at 37 °C and then rinsed twice with PBS and aspirated as described above. All treatment and rinse steps involving dispersin B hydrogel were carried out on ice to maintain the liquid state of the hydrogel. For the second treatment, tubes were filled with 1 mL of PBS; 80 µg/mL dispersin B solution; or 0.1%, 0.5%, 1% or 2.5% BP, as indicated. After 15 min at 37 °C, tubes were rinsed three times with 3 mL of PBS and filled with 1 mL of PBS. Biofilm bacteria were detached from the walls of the tubes by sonication for 30 sec using an IKA Labortechnik sonicator set to 50% power and 50% duty cycle. Control experiments showed that this sonication treatment did not affect the viability of *C. acnes* or *S. epidermidis* cells. Sonicates were serially diluted in PBS. *C. acnes* and *S. epidermidis* CFUs were enumerated independently by plating dilutions on selective media as previously described [29]. To enumerate *S. epidermidis* CFUs, dilutions were plated on TSA and incubated in air which prevented growth of anaerobic *C. acnes*. To enumerate *C. acnes* CFUs, dilutions were plated on TSA supplemented with 20 µg/mL erythromycin and incubated anaerobically which prevented growth of erythromycin-sensitive *S. epidermidi*s.

### 2.6. Crystal violet binding assay

Biofilms were rinsed vigorously with tap water and stained for 1 min with 1 mL of Gram’s crystal violet. Tubes were then rinsed with tap water to remove the unbound dye and air-dried. To quantitate crystal violet binding, tubes were filled with 1 mL of a 33% acetic acid solution, incubated at room temperature for 30 min, and mixed by vortex agitation. A volume of 200 µL of the dissolved dye was transferred to the well of a 96-well microtiter plate and its absorbance at 620 nm was measured in an automated microplate reader. Tubes containing sterile broth were incubated and processed along with the inoculated tubes to serve as controls. The amount of biofilm detachment was quantitated using the formula 1 − (*A*_620/Dispersin B_/*A*_620/No enzyme_) ×  100.

### 2.7. Statistics and reproducibility of results

All experiments were performed in duplicate or triplicate tubes and were repeated three times on different days. All repetitions exhibited a similar range of values and similarly significant differences in mean CFU/tube values among the experimental groups. Representative experiments are shown in the figures. The significance of differences between CFU/tube values was calculated using a two-tailed Student’s *t*-test for pairwise comparisons and a one-way ANOVA with Tukey’s post hoc analysis for comparison of more than two groups. A *p*-value <  0.01 was considered significant.

## 3. Results

### 3.1. Detachment of *C. acnes*/*S. epidermidis* dual-species biofilms by dispersin B solution and dispersin B hydrogel

**Fig 1:**
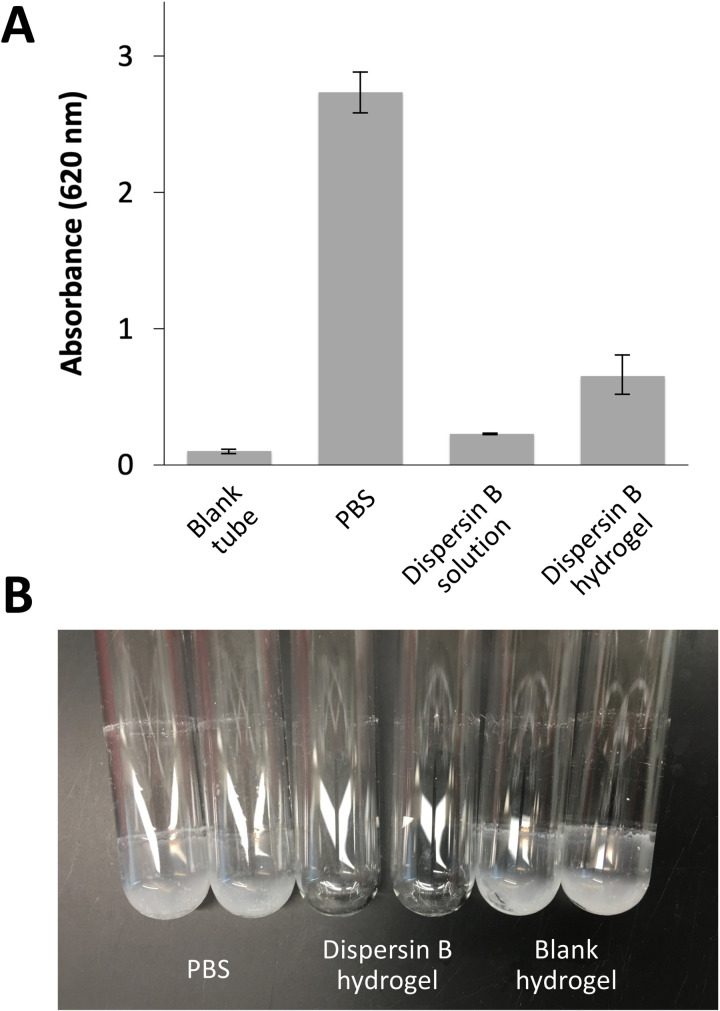
Detachment of *C. acnes*/*S. epidermidis* dual-species biofilms by dispersin B solution and dispersin B hydrogel. Both agents contained 80 µg/mL of enzyme. **(A)** Biofilms were rinsed and treated with the indicated agent for 15 min, then stained with crystal violet dye. The amount of bound dye (Absorbance at 620 nm) is proportional to the amount of biofilm biomass. Control tubes were treated with PBS. Blank tubes contained uninoculated broth. Values show mean and range for triplicate tubes for each condition. **(B)** Detachment of *C. acnes*/*S. epidermidis* dual-species biofilms by dispersin B hydrogel and blank hydrogel (no enzyme). Biofilms were rinsed and treated with the indicated agent for 15 min, then rinsed and photographed. Control tubes were treated with PBS. Duplicate tubes for each condition are shown.

To confirm that dispersin B causes detachment of *C. acnes*/*S. epidermidis* dual-species biofilms grown aerobically in glass tubes, biofilms were treated for 15 min with an 80 µg/mL dispersin B solution, or a hydrogel containing 80 µg/mL dispersin B. The amount of biofilm biomass remaining after treatment was quantitated using a crystal violet binding assay (Fig 1A). Both the dispersin B solution and dispersin B hydrogel treatments caused a significant reduction in biofilm biomass (94% and 78%, respectively). These results are consistent with those of a previous study showing that an 80 µg/mL solution of dispersin B efficiently detached aerobic *C. acnes*/*S. epidermidis* dual-species biofilms from glass tubes [29]. The biofilm detaching activity of dispersin B hydrogel was evidently due to the enzyme and not the other components of the hydrogel because a blank hydrogel (no enzyme) did not cause significant detachment of *C. acnes*/*S. epidermidis* dual-species biofilms as evidenced by visual inspection of the biofilms after treatment (Fig 1B).

### 3.2. Pre-treatment of *C. acnes*/*S. epidermidis* dual-species biofilms with dispersin B solution renders them susceptible to benzoyl peroxide killing

**Fig 2:**
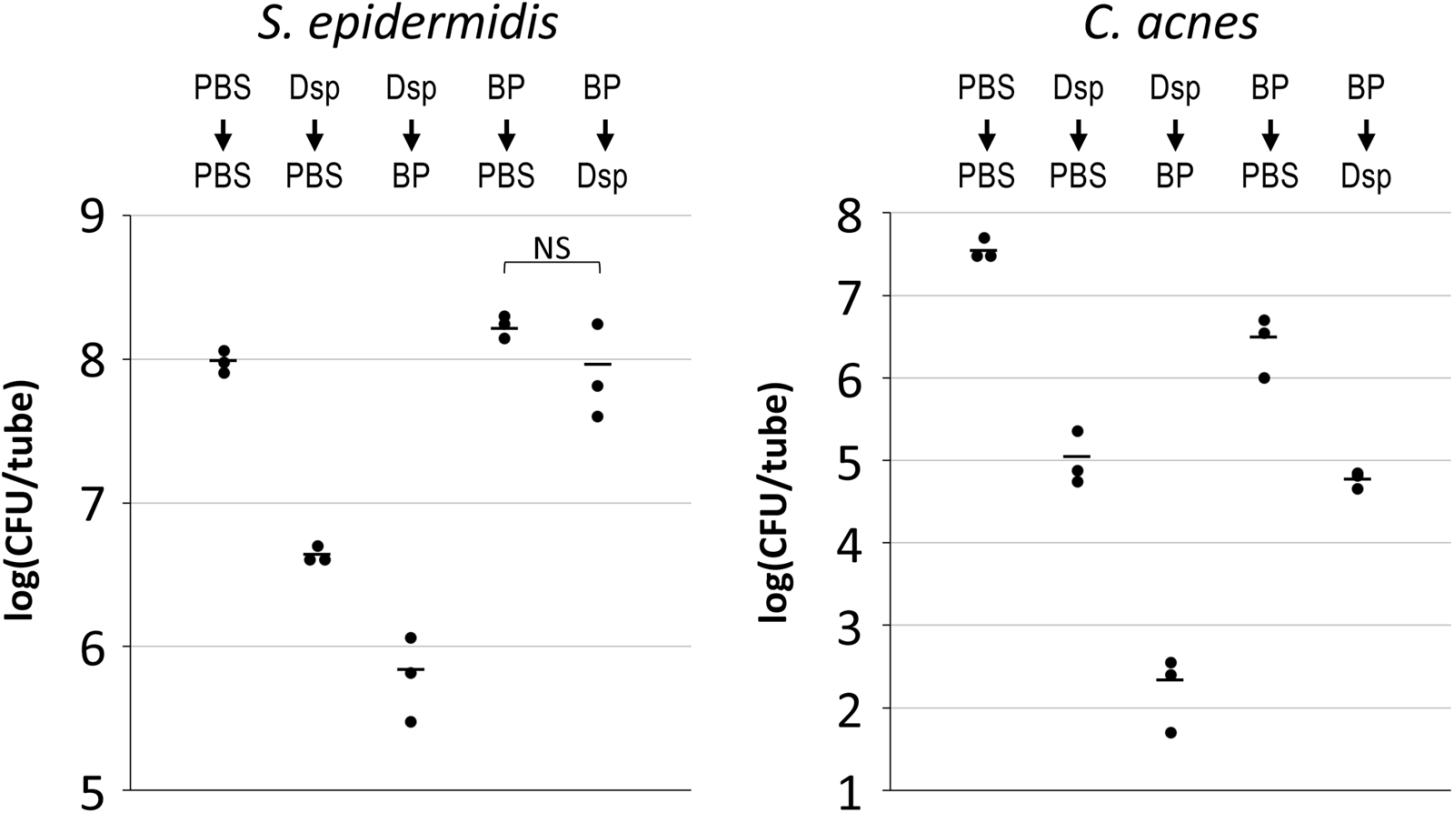
Pre-treatment of *C. acnes*/S*. epidermidis* dual-species biofilms with dispersin B solution renders them sensitive to killing by benzoyl peroxide. 24-h-old aerobic *C. acnes*/*S. epidermidis* dual-species biofilms were rinsed and treated for 15 min with phosphate buffered saline (PBS), 80 µg/ml dispersin B solution (Dsp), or 1% benzoyl peroxide (BP), and then rinsed and subjected to a second 15-min treatment with PBS, Dsp or BP in the combinations indicated at the top. Graphs show CFU/tube values for *S. epidermidis* (left panel) and *C. acnes* (right panel) from triplicate tubes for each treatment. Each dot represents one tube. Horizontal bars indicate means. NS, not significantly different.

*C. acnes*/*S. epidermidis* dual-species biofilms were subjected to a series of two-step treatments consisting of PBS, 80 µg/mL dispersin B solution, or 1% BP in different combinations. After treatment, biofilms were rinsed to remove loosely adherent cells, and then detached from the walls of the tube by sonication. *S. epidermidis* and *C. acnes* CFUs were enumerated independently by plating on selective agar (Fig 2). Dispersin B treatment alone caused a >1 log reduction in *S. epidermidis* CFUs and a >2 log reduction in *C. acnes* CFUs. Treatment of biofilms with 1% BP alone caused no reduction in *S. epidermidis* CFUs and a 1 log reduction in *C. acnes* CFUs. However, treatment of biofilms with dispersin B followed by BP caused a >2 log reduction in *S. epidermidis* CFUs and a >5 log reduction in *C. acnes* CFUs. These results suggest that dispersin B sensitizes both *S. epidermidis* and *C. acnes* to BP killing when cultured aerobically in a dual-species biofilm. Treatment of biofilms with BP followed by dispersin B resulted in no significant detachment or killing of *S. epidermidis*, suggesting that residual BP may partially inactivate or inhibit dispersin B (Fig 2, left panel). BP treatment followed by dispersin B treatment resulted in a 3-log reduction in *C. acnes* CFUs compared to a >5 log reduction when biofilms were treated with dispersin B followed by BP (Fig 2, right panel).

**Fig 3:**
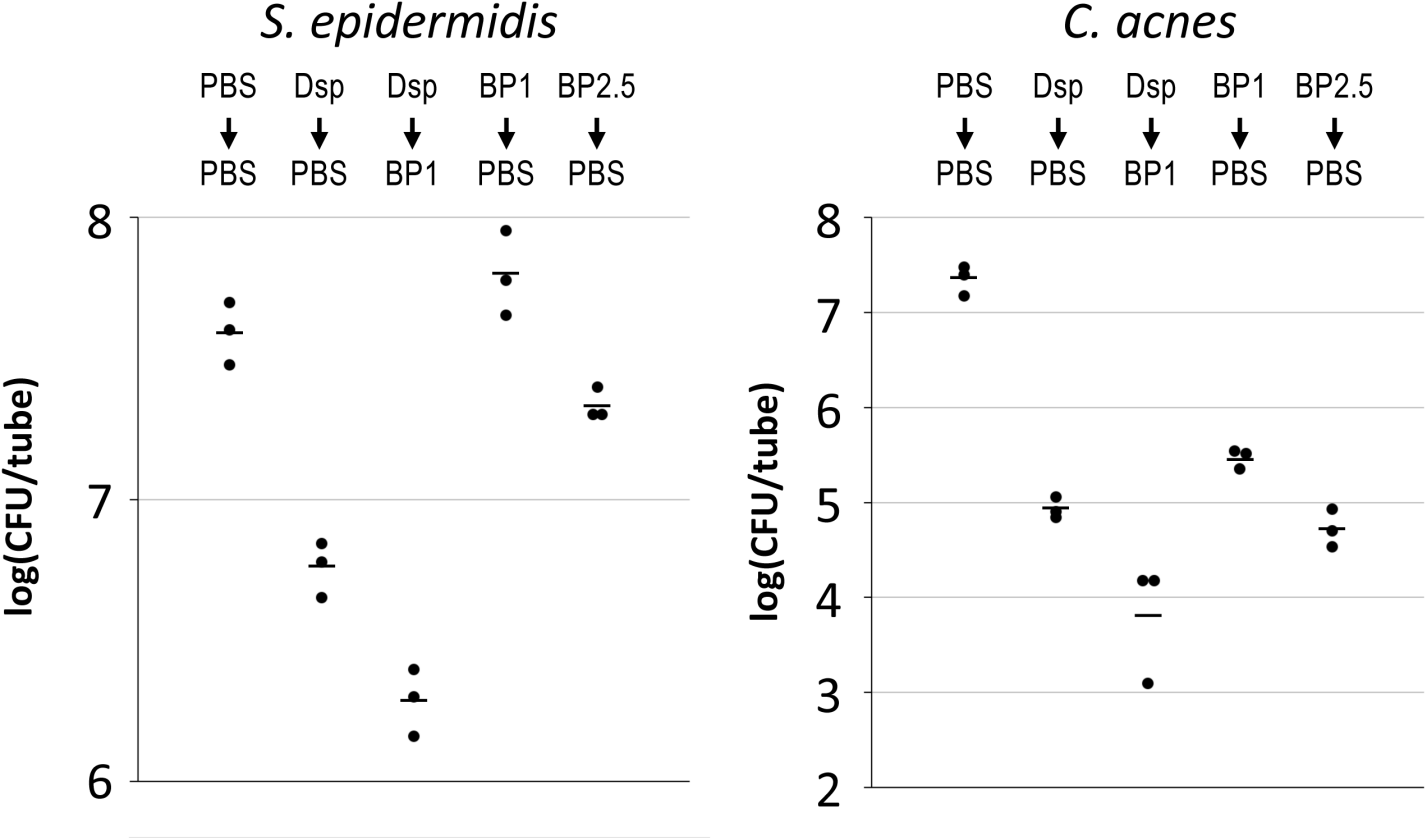
Dispersin B solution +  1% benzoyl peroxide kills *C. acnes* biofilms as efficiently as 2.5% benzoyl peroxide alone. 24-h-old aerobic *C. acnes*/*S. epidermidis* dual-species biofilms were rinsed and treated for 15 min with phosphate buffered saline (PBS), 80 µg/ml dispersin B solution (Dsp), or 1% or 2.5% benzoyl peroxide (BP1 and BP2.5, respectively), then rinsed and subjected to a second 15-min treatment with PBS or BP1 in the combinations indicated at the top. Graphs show CFU/tube values for *S. epidermidis* (left panel) and C*. acnes* (right panel) from triplicate tubes for each treatment. Each dot represents one tube. Horizontal bars indicate means.

In a similar experiment, treatment of dual-species biofilms with dispersin B followed by 1% BP resulted in levels of *C. acnes* detachment and killing equivalent to those observed with 2.5% BP alone (Fig. 3). These findings suggest that dispersin B treatment may lower the minimum effective dose of BP against *C. acnes*.

### 3.3. Dispersin B hydrogel acts synergistically with benzoyl peroxide to kill *C. acnes* in *C. acnes*/*S. epidermidis* dual-species biofilms

**Fig 4:**
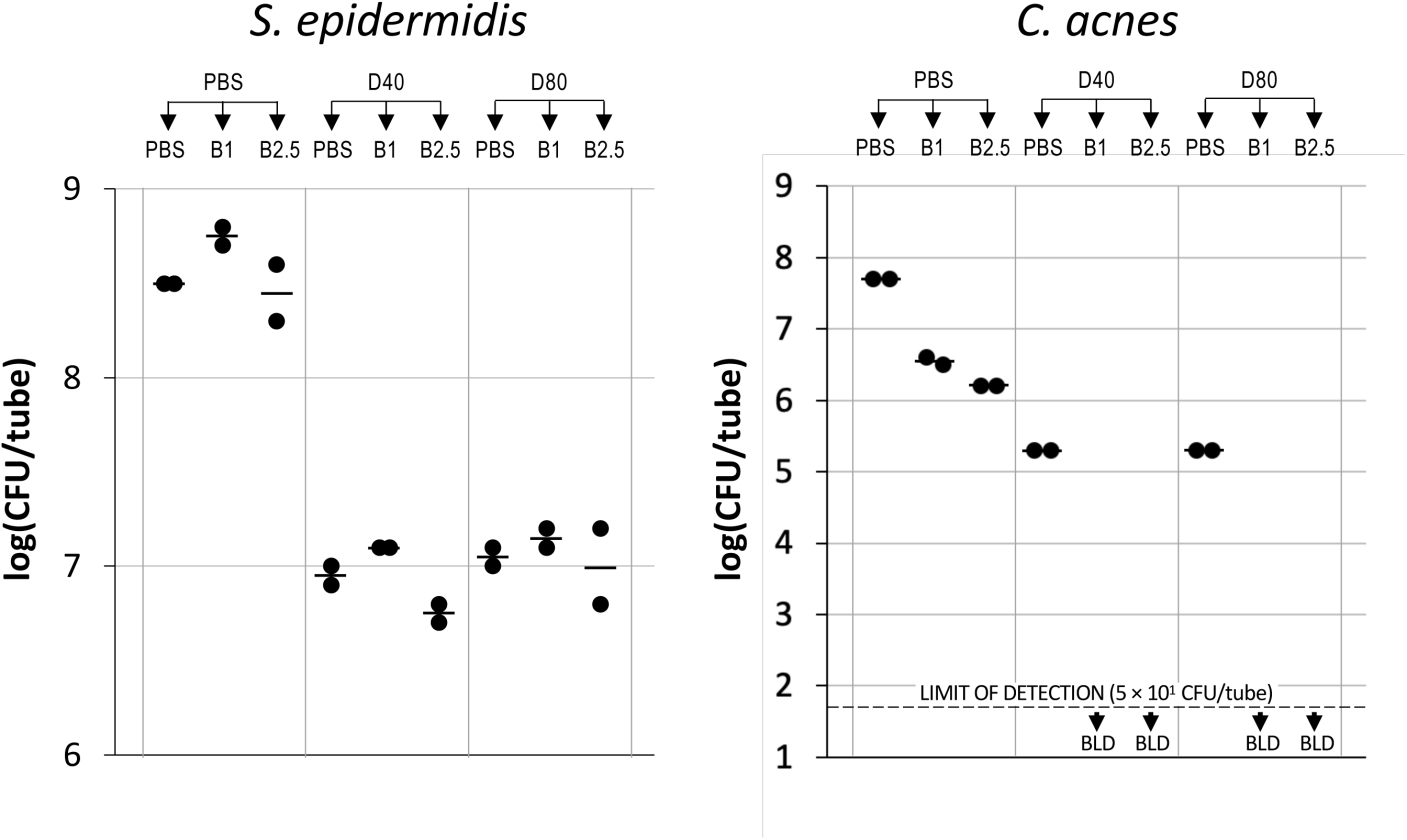
Dispersin B hydrogel sensitizes *C. acnes* biofilms to benzoyl peroxide killing. 24-h-old aerobic *C. acnes*/*S. epidermidis* dual-species biofilms were rinsed and treated for 15 min with phosphate buffered saline (PBS) or dispersin B hydrogel containing 40 or 80 µg/mL dispersin **B** (D40 and D80, respectively), then rinsed and subjected to a second 15-min treatment with PBS or benzoyl peroxide at 1% or 2.5% (B1 and B2.5, respectively), in the combinations shown above. Graphs show CFU/tube values for *S. epidermidis* (left panel) and *C. acnes* (right panel) from duplicate tubes for each treatment. Dashed line in the right panel indicates the limit of detection (5 ×  101 CFU/tube). Each dot represents one tube. Horizontal bars indicate means. BLD, below limit of detection.

To determine whether dispersin B hydrogel can sensitize *C. acnes*/*S. epidermidis* dual-species biofilms to BP killing, biofilms were subjected to a two-step treatment where the first step consisted of PBS, or dispersin B hydrogel containing 40 or 80 µg/mL dispersin B, and the second step consisted of PBS, 1% BP, or 2.5% BP. After treatment, biofilms were rinsed to remove dispersed cells, then detached from the walls of the tube by sonication. *S. epidermidis* and *C. acnes* CFUs were enumerated independently by plating on selective agar (Fig 4). Dispersin B hydrogel alone at 40 or 80 µg/mL resulted in a >1 log reduction in *S. epidermidis* CFUs, while BP alone at 1% or 2.5% exhibited no significant killing of *S. epidermidis* biofilms (Fig 4, left panel). Dispersin B hydrogel at 40 or 80 µg/mL did not sensitize *S. epidermidis* to 1% or 2.5% BP killing under these conditions (Fig 4, left panel). In contrast, dispersin B hydrogel alone at 40 or 80 µg/mL resulted in a >2 log reduction in *C. acnes* CFUs, BP alone at 1% or 2.5% caused >1 log killing of *C. acnes* biofilms, and dispersin B hydrogel at 40 or 80 µg/mL followed by BP at 1% or 2.5% caused >6 log reduction in *C. acnes* CFUs (Fig 4, right panel). These results suggest that dispersin B hydrogel acts synergistically with BP to kill *C. acnes* in *C. acnes*/*S. epidermidis* dual-species biofilms.

**Fig 5:**
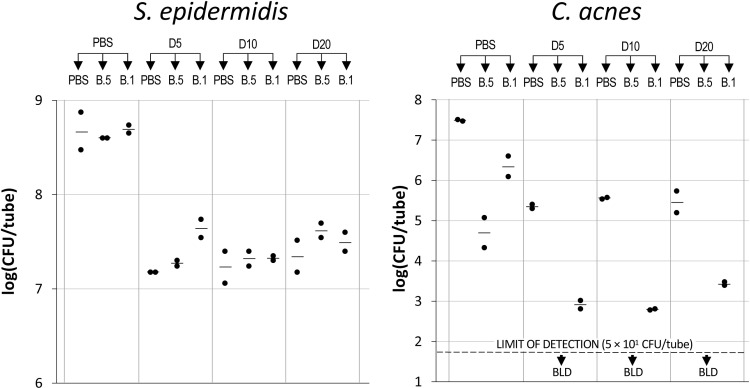
Dispersin B hydrogel sensitizes *C. acnes* biofilms to benzoyl peroxide killing. 24-h-old aerobic *C. acnes*/*S. epidermidis* dual-species biofilms were rinsed and treated for 15 min with phosphate buffered saline (PBS) or dispersin B hydrogel containing 5, 10 or 20 µg/mL of enzyme (D20, D10, and D5 respectively), and then rinsed and treated with PBS or benzoyl peroxide at 0.5% or 0.1% (B.5 and B.1, respectively). Graphs show CFU/tube values for *S. epidermidis* (left panel) and *C. acnes* (right panel) from duplicate tubes for each treatment. Each dot represents one tube. Horizontal bars indicate means. BLD, below limit of detection.

To determine the lowest concentrations of dispersin B and BP needed to eradicate *C. acnes* from *C. acnes*/*S. epidermidis* dual-species biofilms, biofilms were treated with dispersin B hydrogel containing 20, 10 or 5 µg/mL dispersin B, followed by BP at 0.5% or 0.1% (Fig 5). Dispersin B hydrogel alone at enzyme concentrations of 20, 10 or 5 µg/mL resulted in a >1 log reduction in *S. epidermidis* CFUs and a 2-log reduction in *C. acnes* CFUs (Fig 5), while benzoyl peroxide alone at 0.5% or 0.1% exhibited no significant killing of *S. epidermidis* biofilms (Fig 5, left panel) and a 1–2 log reduction in *C. acnes* CFUs (Fig 5, right panel). Dispersin B hydrogel at 20, 10 or 5 µg/mL of enzyme did not sensitize *S. epidermidis* to killing by 0.5% or 0.1% BP (Fig 5, left panel). In contrast, treatment of biofilms with dispersin B hydrogel at enzyme concentrations 20, 10 or 5 µg/mL resulted in a >3 log increase in *C. acnes* killing by both 0.5% and 0.1% BP compared to 0.5% and 0.1% BP alone (Fig 5, right panel).

## 4. Discussion

Benzoyl peroxide (BP) has remained a cornerstone of acne treatment since its introduction in the 1950s. As an oxidizing agent, BP non-specifically oxidizes bacterial surface proteins, leading to structural damage and rapid cell death. BP’s main advantages include its ability to circumvent antibiotic resistance, as it is effective against antibiotic-resistant strains and has not been shown to induce resistance in *C. acnes*. However, a significant drawback of BP is its skin irritability, which often leads to patients using lower doses to reduce irritation. Unfortunately, these reduced doses may be less effective because they require longer contact times to kill *C. acnes* [33].

Previous research has demonstrated the microbiocidal effects of BP against *C. acnes* and *S. epidermidis in vitro* [34,35]. However, a study by Zhou et al. [36] observed that, following 12 weeks of BP treatment, the prevalence of *C. acnes* decreased, while that of *S. epidermidis* increased in the skin microbiota of acne patients. In our study, we observed that BP concentrations ranging from 0.1% to 2.5% effectively killed *C. acnes* but did not affect *S. epidermidis* when both bacteria were co-cultured in aerobic biofilms *in vitro.* This differential susceptibility to BP could be attributed to variations in bacterial strains, experimental conditions, or the complexities of the *in vivo* environment versus *in vitro* models.

Dispersin B has been shown to sensitize anaerobic *C. acnes* mono-species biofilms to BP and tetracycline [24] and to disperse aerobic *C. acnes*/*S. epidermidis* dual-species biofilms in glass tubes [29]. In this study, we demonstrated that dispersin B also sensitizes aerobic *C. acnes*/*S. epidermidis* dual-species biofilms to BP-induced killing. These findings are consistent with the biocide-sensitizing effects of dispersin B observed against other bacterial species [26,37–39]. Due to its high specificity for PNAG [40], these results suggest that PNAG plays a crucial role in the tolerance of *C. acnes* to BP in aerobic dual-species biofilms. The use of dispersin B in combination with BP could therefore be a promising strategy for acne treatment and prevention, as it may reduce the minimum effective dose of BP and consequently mitigate skin irritation.

Our study underscores the critical role of PNAG in protecting *C. acnes* from BP killing in the context of aerobic dual-species biofilms with *S. epidermidis*. However, a key unresolved question remains: the source of the PNAG in these biofilms. It is well-documented that *S. epidermidis* strain 5 produces PNAG [32], but it remains unclear whether *C. acnes* strain HL086PA1 produces PNAG as well. Previous studies have shown that all four *C. acnes* strains tested to date produce PNAG [24], suggesting that *C. acnes* PNAG might also contribute to the observed BP resistance. Further experiments are needed to determine whether PNAG production in the aerobic *C. acnes*/*S. epidermidis* dual-species biofilms arises from one or both bacterial species, which could provide valuable insights into how PNAG-mediated resistance functions in this context.

A key limitation of the present study is the artificial nature of the *in vitro* biofilm model used. Biofilms were formed on the surface of glass tubes in Tryptic Soy broth (TSB), which does not fully replicate the lipid-rich environment of the skin, where *C. acnes* naturally resides. *In vivo*, *C. acnes* biofilms are likely influenced by sebum composition, host immune factors, and tissue interactions that are absent in our experimental conditions. Future studies should explore models that incorporate lipid-rich environments and more physiologically relevant conditions to better mimic the complexity of *C. acnes* biofilms in the skin.

## Abbreviations

BP: benzoyl peroxide
CFU: colony-forming unit
PBS: phosphate buffered saline
PNAG: poly-*N*-acetylglucosamine
TSA: Tryptic Soy agar
TSB: Tryptic Soy broth.
